# Enhancing physical healthcare in the mental health system: Perspective from the 2024 Equally Well Forum Embedding Lived Experience

**DOI:** 10.1177/00048674251387867

**Published:** 2025-10-30

**Authors:** Justin Chapman, Victoria Erskine, John Allan, Dave Peters, Russell Roberts

**Affiliations:** 1 Equally Well, Faculty of Business Justice and Behavioural Science, Charles Sturt University, Bathurst, NSW, Australia; 2 School of Pharmacy and Medical Science, Centre for Mental Health, Griffith University, Brisbane, QLD, Australia; 3 Addiction and Mental Health Service, Metro South Health, Brisbane, QLD, Australia; 4 Faculty of Medicine, University of Queensland, Brisbane, QLD, Australia

**Keywords:** Systems reform, mental health, mental illness, experiential evidence, advocacy, priority setting

## Abstract

Each year in Australia, tens of thousands of people living with mental illness die from preventable physical health conditions. Australia is yet to meet its 2008 commitment to provide equal healthcare for people with disabilities (Article 25, United Nations Convention on the Rights of Persons with Disabilities). In May 2024, a national forum of 240 delegates called for urgent action to address the life expectancy gap for people living with mental illness. This article presents perspectives of forum delegates about: (1) *where we need to be*, (2) *critical reform elements* and (3) *foundational components* to address this health inequity. Attendees overwhelmingly expressed the need for lived experience leadership and human rights to underpin the health system. Foundational components included respectful and inclusive practices, co-learning and co-production, a strong and integrated lived experience workforce, strategic support for reform, and societal shifts in values. Recommendations for government, advocacy bodies, services and individuals were provided, including conducting analyses of mental health legislation to align with Article 25 of the United Nations Convention on the Rights of Persons with Disabilities; establishing strategic lived experience leadership positions; providing workforce training on the increased risk of premature death and human rights approaches to improving healthcare equity; and taking every opportunity to advocate for physical health equality for people with mental illness. Urgent public health action is needed to incentivise and instil accountability for change, ensuring that reform efforts add power to the voices of those most impacted by mental illness and the services designed to support them.

## Introduction

The poor health and increased risk of premature death of the 5 million Australians living with mental illness is arguably the most pressing national public health challenge. This mortality gap can vary depending on the type and severity of illness: the life expectancy gap is closer to 13 years for people with higher prevalence conditions such as depression, 14 years for people with severe mental illness and 20 years for substance use disorders (Chan et al., 2023). Recent Australian figures indicate an 11-year life expectancy gap for persons who accessed federally funded mental health-related services nationally (Roberts et al., 2024). Significantly, this analysis revealed that almost two thirds of these deaths were ‘excess’, with over 16,000 deaths under the age of 75 years each year being potentially preventable (as defined in Organisation for Economic Co-operation and Development (OECD), 2021). The early mortality of people living with a mental illness is recognised internationally as a breach of human rights (Thornicroft, 2011), and is indicative of Australia’s failure to honour its 2008 commitment to the United Nations Convention on the Rights of Persons with Disabilities (UNCRPD), specifically in relation to Article 25 (UN General Assembly, 2006).

Recognising the need for action, in 2016 the *National Mental Health Commission* supported a coalition of health service providers, organisations, consumer and advocacy groups and researchers to develop a statement of priorities for reform. The resulting *Equally Well Consensus Statement* outlined 48 actions across six Essential Elements (National Mental Health Commission, 2016): (1) a holistic, person-centred approach; (2) effective promotion, prevention and early intervention; (3) equity of access; (4) improved quality of healthcare; (5) care coordination and regional integration; and (6) monitoring progress. Using a collective impact approach (Kania et al., 2022), Equally Well works to coordinate, activate and support initiatives to reduce the life expectancy gap between people living with mental illness and the rest of the population. It does this by facilitating connection and collaboration across 100 supporting organisations who have committed to this common goal.

The Equally Well consensus statement was adopted in the *Fifth National Mental Health and Suicide Prevention plan 2017-2022*, Priority Area 5: Improving the physical health of people living with mental illness and reducing early mortality (Department of Health, 2017). Furthermore, the 2*020 Productivity Commission’s Inquiry into Mental Health* recommended improving physical health as a ‘start now’ and a ‘priority’ reform (Productivity Commission, 2020). The 2023 *Equally Well in Action* report summarised 308 initiatives implemented to improve the physical health of people living with mental illness across Australia (Department of Health and Aged Care, 2022). As part of an ongoing national data linkage monitoring strategy, the first *Mortality of people using Australian Government-funded mental health services and prescription medications* report demonstrated that people who access mental health-related treatments have an 11-year shorter life expectancy, and 60% of this mortality gap is potentially preventable (Roberts et al., 2024). Regular symposia on the physical health of people living with mental illness are a mechanism for sharing research, facilitating collaboration and public engagement to address this public health problem. Building on 7 years of momentum, this article presents a summary of the perspectives and resolutions of delegates at this forum.

The 2024 National Equally Well Forum: Embedding Lived-Experience was held from 27th to 28th of May in Melbourne, Australia. The focus of the forum was to elevate the lived experience voice aligned with Equally Well’s vision and aim, with the goal of developing a Call to Action to provide a new direction for the movement. As an openly accessible forum promoted through Equally Well networks, 240 delegates from a range of backgrounds (Table 1) attended panels and plenary sessions, and participated in facilitated workshops each with a pre-assigned Chair and scribe. Forum recordings were descriptively analysed by Equally Well staff, reviewed by the Equally Well Alliance, and a draft summary was circulated to Forum attendees via email. The draft summary was presented to delegates in a webinar along with a questionnaire inviting further input. The outcome was a Call to Action to support individuals, services, advocacy bodies and governments to address this systemic public health challenge.

**Table 1.** Profile of delegates.

Self-reported identification	*n*
Consumer	71
Carer	22
Clinician	50
Service Provider	16
Policy Maker	20
Researcher/Educator	33
Other	28
Total	240

This Perspective article provides an overview of the recommendations arising from the forum, presented in three sections: (1) *Where we need to be*, (2) *Critical reform elements* and (3) *Foundational components*. Direct quotes are provided to reflect the diverse perspectives from participants at the event.

### Where we need to be

Physical health was recognised as integral to the wellbeing of people living with mental illness. Forum attendees emphasised that contextual and relational approaches need to be fundamental throughout health, human and social service systems to improve community health and wellbeing. Contextual and relational elements include the social environment, opportunities, relationships, community roles, and interactions with society and services. The current healthcare paradigm is largely based in biomedical and individualistic approaches, which minimises these other essential elements of health and wellbeing:

*If we don’t understand the social determinants of health, we’re not going to get it right.*

There was a consistent view that fundamental shifts are needed to ‘invert’ the system from being reactive and reductionistic to wellbeing-promoting and holistic. This vision of a more human-centred system was articulated in many ways by lived experience leaders and forum attendees, with frequent reference to First Nations conceptualisations of social and emotional wellbeing (Department of the Prime Minister and Cabinet, 2017). This would lead to a system where social and emotional wellbeing needs are addressed as a priority, with biomedical and psychological treatments being essential adjuncts to support the recovery journey:

*Health is a fundamental human right, the foundation for all the freedoms – without health, the pursuit of happiness, equality and justice crumbles.*

Yet most families, carers and kin feel they are ignored and excluded from care and care planning (Kaine et al., 2022). Family and carers often have decades of experience and devote many hours each week to caring and supporting their family member, thus their knowledge and expertise deserves much greater respect and recognition as partners in providing care:

*We need to change the mindset of health professionals to see the whole person and the relationships and context of the person – we need systems change to recognise and value carers and family – not ignoring or excluding.*

Improving wellbeing by adequately addressing social determinants and relationships was seen as pivotal to improving physical health outcomes. There was broad consensus that the people who are most impacted by services must be leading and directing efforts to reform the system towards this vision.

### Critical reform elements

Human rights and lived experience were described as the two critical elements needed to underpin system reform efforts. Health inequality and poor physical health outcomes were seen as symptoms of a system in dire need of reform, caused by inadequate adoption of human rights legislation and lived experience centrality:

*Human rights are the foundation of lived experience leadership – it transforms personal stories into calls for justice and equality. Lived experience leadership is the heartbeat of human rights – it transforms abstract principles into tangible outcomes and meaning.*

The urgent need for human rights to underpin healthcare systems and practices rather than being an afterthought was emphasised throughout the forum. Reform to align mental health legislation with Article 25 of the UNCRPD to ensure equitable access to quality physical healthcare was highlighted as a priority:

*Despite Australia signing the UNCRPD at a federal level, we have largely failed to convert Mental Health Acts to be consistent with it.*

The lived experience movement has instigated substantial shifts in our knowledge construction of mental illness and recovery. Authentic centring of lived experience throughout all levels of our healthcare systems is needed to ensure that human rights frameworks are appropriately understood and applied:

*We have a long way to go – the past highlights the courage and bravery and the new ground and ‘mould breaking’ that lived experience is delivering, and upon which we’ll need to continue drawing on.*

These critical reform elements will guide us towards a more humanistic system that facilitates improvements in wellbeing and resilience and minimises harm for the people and communities they are designed to serve. A vision was provided for the future:

*Every time someone has contact with a service, they ask about your holistic health, there’s no discrimination because of a history of mental illness or addiction, and you feel good, and think ‘they’ve heard me, and they’re on my side’.*

### Foundational components

**Figure 1.**
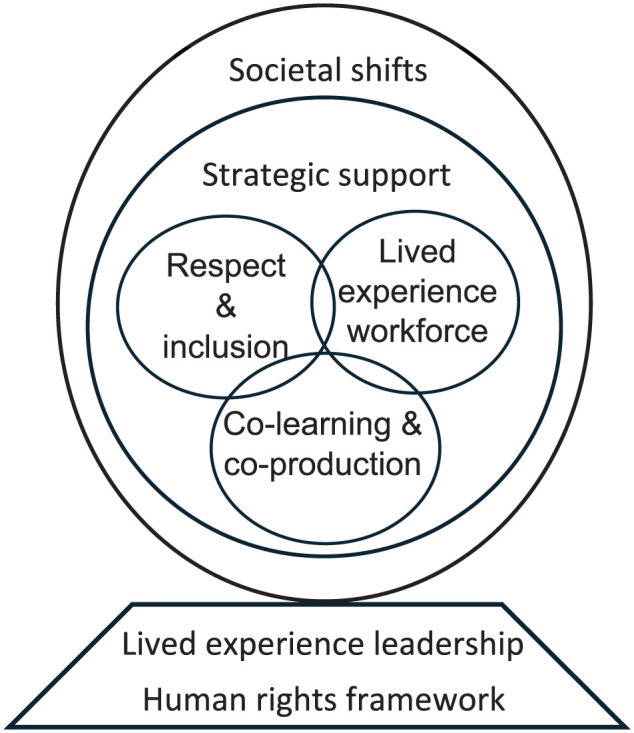
Critical reform elements and key ingredients needed to progress towards a more humanistic system in which contextual and relational elements of health and wellbeing are fundamental throughout our health, human and social service systems.

Critical reform elements must underpin the change process, and target different components of the system, including its values, skills, workforce, service models and the broader societal context. Five *foundational components* emerged from reviewing contributions made by forum delegates (Figure 1): (1) respect and inclusion; (2) co-learning and co-production; (3) lived experience workforce; (4) strategic support; and (5) societal shifts.

***Respect and inclusion*** are fundamental values that underpin a service system focussed on equity of health outcomes. A lack of inclusion often stems from a lack of understanding, which can be addressed through lived experience and human rights framings. More inclusive practices require greater appreciation of diverse identities and the contextual and relational influences important to wellbeing for these different groups:

*. . . providing inclusive care takes time, resources and energy. Workforce burnout and high staff turnover contributes to less inclusive practices.*

There are many groups whose voices are not heard and respected, such as people with lived experience, their families and carers, First Nations people, women, LGBTQI+ people and migrants, including the people working within the system doing the best they can for the communities they serve. Systemic exclusion can instil fear in those marginalised, leading to disengagement and poorer health outcomes:

*There’s a lot to say about intersectionality – the more open and diverse views we have, particularly in lived experience, the more likely we’ll get it right.*

Training and education in these areas may improve knowledge and awareness leading to improvements in practice; however, more innovative approaches are needed to embed these values into the system.

***Co-learning and co-production*** approaches are needed for inclusive practices and human rights framings to be embodied in organisational culture. Co-learning involves collaborative and reflective practices with all relevant parties in a non-hierarchical manner, and co-production involves lived experience leadership in defining the problem, designing and delivering the solution and evaluating the outcome (Groot et al., 2022).

Safer Care Victoria presented on the Mental Health Improvement Program which operates on a ‘tripartite’ model involving clinical and non-clinical workforce teams, consumers and their families, carers and supporters to develop workforce capability and implement quality improvement. Education and training should be cross-referential: *‘We need to learn from each other in the tripartite arrangement including consumers, families and clinicians’*.

Co-learning should be embedded into every level of service planning, policy and practice, including care planning and care review process, and co-production should be embedded into workforce development, quality improvement, regional planning and policy development. Integrating co-learning and co-production approaches across multiple levels of the system would help identify gaps in healthcare access and embed respect and inclusion into routine practice.

The ***lived experience workforce*** can play an important role in changing organisational culture and providing human-centred care for people living with mental illness. Forum attendees frequently emphasised that a lived experience workforce can extend practice from clinically oriented paradigms to encompass more holistic approaches, as well as augment the existing mental health workforces:

*We need to be working in our community, not in mental health services. We should be co-facilitating with clinicians which is also a good opportunity to educate clinicians about what works for people with mental health issues.*

Specific supportive roles for peers were suggested, such as physical healthcare navigators in General Practice clinics and multi-disciplinary mental health teams, and helping with advocacy and coordination of physical health and chronic care appointments. The non-clinical nature of peer support was seen as an advantage and synergistic with humanistic and holistic values that can promote physical health. The necessity for these roles to be properly supported through peer mentoring and supervision was emphasised.

***Strategic support*** from decision-makers is needed to facilitate these shifts. Forum attendees acknowledged that government support needs to *add* power to the voices of those most impacted by mental illness and the services that strive to support them. This involves supporting stakeholders and lived experience leadership in developing policies and strategies, and timely enactment of policy levers to incentivise change and instil accountability (Grace et al., 2015):

*The decision-making about how we spend taxpayers’ money on healthcare reform should be guided by people who understand the value of lived experience and mental health.*

The lived experience movement was emphasised as the catalyst for change: ‘*we can go towards transformation, which is why lived experience leadership is such a much-needed concept*’. With strategic support, lived experience leadership positions and ‘grassroots’ mechanisms for engaging people with lived experience can embed human rights framings into continued systems change processes. This must be at all levels of our healthcare services, beginning at strategic levels of legislation, accreditation standards, governance structures and models of care:

*Models of care won’t transform culture unless they are properly designed with using human rights analysis, then we see trauma informed, recovery-oriented care emerges from this foundation.*

The role of research and evaluation in guiding systems change was seen as essential. However, it was stressed that experiential evidence from people with lived experience should be prioritised and considered ‘*on the same par*’ as other scientific evidence. At a broader societal level, this depends on greater awareness about mental health challenges and recognition of lived experience as expertise.

***Societal shifts*** are needed to eliminate systemic prejudice and discrimination towards people living with mental illness. Many of the presentations and personal accounts shared at the forum involved experiences of discrimination during healthcare interactions which resulted in significant harm. Prejudice and discrimination exist at individual, institutional and structural levels, and is a root cause of health inequality and healthcare inequity experienced by people living with mental illness:

*The core issue is this failure to see people with lived experience holistically, which is a problem in terms of physical health because people are just seen as a having a mental health issue.*

Terms such as ‘diagnostic overshadowing’ and ‘stigma’ have previously been used to describe this healthcare disparity; however, more explicit terminology was encouraged: ‘*We need to call-out mistreatment for what it is – prejudice and discrimination. This places the lived experience movement more squarely into a rights-based framework*’. While specific healthcare reforms may improve the healthcare system, eliminating prejudice and discrimination requires fundamental shifts in our social fabric. For health equality and healthcare equity to be truly realised, respect and inclusion needs to be foundational in our social values, norms, culture and structures:

*The environment must change, so that things that are currently acceptable are no longer acceptable – that’s our vision for a better understanding and relationship with mental health and addiction.*

It was noted that progress in this area has been hindered because of inaction, with three key action items related to rectifying discrimination from the 5th National Mental Health and Suicide Prevention Plan remaining undelivered (Actions 19.1, 26, 27).

## Conclusion

The premature mortality and high rates of preventable disease experienced by people living with mental illness is a breach of the UNCRPD (UN General Assembly, 2006). Ratified by Australia in 2008, the convention states that Parties (to the convention) ‘*shall provide persons with disabilities with the same range, quality and standard of free or affordable health care and programmes as provided to other persons*’, including prevention and early intervention of preventable chronic diseases. Critical reform elements of human rights and lived experience must underpin progress towards this commitment.

The resolutions arising from this Forum should be considered in concert with the Equally Well National Consensus Statement, and previously published recommendations to protect and improve the physical health of people with mental illness (Roberts et al., 2022, 2025). These include: increasing uptake of available smoking cessation, cancer screening and vaccination programmes; incentivising integrated physical and mental healthcare; embedding physical healthcare nurses and general practitioners in mental health services; establishment of Physical Health Care Navigators; the reconsideration of national prevention programmes (heart disease, respiratory disease, cancer, diabetes and infectious diseases); and establishing a national strategy and implementation plan. This Perspective presents a high-level summary of the collective contributions of forum delegates, and does not represent all individual perspectives. Likewise, it is not presented as the official perspective of the >100 organisations and professional groups who formally support the Equally Well National Consensus Statement.

The Call to Action arising from the 2024 Equally Well Forum outlines 13 priorities for governments, advocacy bodies, services and individuals. Recommendations for government include conducting analyses of mental health legislation to align with Article 25 of the UNCRPD, and establishing strategic lived experience positions including a Chief Lived Experience Officer. Recommendations for advocacy bodies include elevating physical healthcare into planning, commissioning and service improvement, and developing physical health key performance indicators. Recommendations for services include providing workforce training on the increased risk of premature death for people with mental illness and human rights approaches to improving healthcare equity, and establish ‘tripartite’ co-learning processes involving consumers, clinical and non-clinical staff and families, carers and kin. Recommendations for individuals included advocating for physical health equality for people with mental illness through Equally Well’s advocacy activities and resources.

Systemic reform must be founded on affirmative action, respect and strategic support to add power to lived experience voices, targeting changes in legislation, accreditation standards, monitoring, governance and commissioning strategies across federal and state funded systems. While further research is always needed, there is more than sufficient research demonstrating poor physical health outcomes of people with mental illness – the missing element is coordinated and collective advocacy. This Call to Action summons all levels of the community, healthcare services and government to coordinated action towards improving health equality and reducing the unacceptable life expectancy gap for people living with mental illness. The full Call to Action and associated reports will be available on the Equally Well website (Equally Well, 2025).
